# Nutraceuticals to Mitigate the Secret Killers in Animals

**DOI:** 10.3390/vetsci9080435

**Published:** 2022-08-16

**Authors:** Guillermo Tellez-Isaias, Wolfgang Eisenreich, Awad A. Shehata

**Affiliations:** 1 Department of Poultry Science, University of Arkansas Agricultural Experiment Station, Fayetteville, AR 72701, USA; 2 Department Chemie, Technische Universität München, Lichtenbergstraße 4, 85747 Garching, Germany; 3 Avian and Rabbit Diseases Department, Faculty of Veterinary Medicine, University of Sadat City, Sadat City 32897, Egypt; 4 Research and Development Section, PerNaturam GmbH, 56290 Gödenroth, Germany; 5 Prophy-Institute for Applied Prophylaxis, 59159 Bönen, Germany

In the past few years, the concept of “gut health” has established itself as a norm in the scientific literature and animal production. Although the term “gut health” is not specially well-defined, scientists are in agreement that it refers to the capacity of the gastrointestinal tract (GIT) to carry out normal physiological processes and to maintain homeostasis, thereby enabling it to withstand both infectious and non-infectious stressors.

It is believed that persistent inflammation in the colon is the basic cause of ninety percent of all disorders [1]. The host’s biology, metabolism, nutrition, immunology, and neuroendocrine system are all profoundly influenced by the microbiota that live in the gut [2,3]. Short-chain fatty acids, gastrointestinal hormones, enteroendocrine cells, and immune cells all play a role in the mediation of these effects [4]. The enteric neural system and hormonal networks are responsible for controlling GIT motility; however, these systems are disrupted in functional GIT disorders [5]. The delicate intestinal epithelial barrier is significantly influenced by the neuroendocrine network that links the central nervous system, enteric nervous system, intestinal microbiota, and the GALT [6]. This network connects the four previously mentioned systems. This barrier, which consists of a single layer of enterocytes with strong intercellular connections, is responsible for regulating the appropriate level of tolerance and immunization in response to antigens that are not self-producing [7].

Therefore, the function of the gastrointestinal tract is absolutely necessary for preserving a proper equilibrium between health and illness. Both chronic stress and chronic intestinal inflammation take a large amount of the body’s resources away from normal growth and reproduction in order to keep this system in survival mode. Perhaps a more all-encompassing definition of “gut health” might include the harmonious interactions within the microbiota–brain–gut axis [8], which describes the relationship between these three factors.

The many microbiomes that reside on mucosal surfaces are in equilibrium throughout all biological and physiological processes [9]. The loss of intestinal integrity can be attributed to dysbiosis, which occurs when the microbiota in the GIT go out of balance [10]. The components of the diet as well as the viscosity of the contents of the gut have an effect on the microbes that live in the small intestine [11]. The body’s response to cellular injury and the culmination of the stress response process is known as inflammation. This response occurs independently of the kind or origin of the injury (biological, environmental, nutritional, physical, chemical, or psychological).

Inflammation and stress are both examples of nonspecific defense mechanisms known as innate reactions. These responses involve hormones, neuropeptides, immune cells, and molecular mediators, all of which are crucial for the processes of survival and recovery in all forms of life. The autonomic nervous system and the endocrine hormones (adrenaline and glucocorticoids) work together to prepare every single cell in the body for either “fight or flight” when the body is exposed to a stressful scenario. If the stressful signal continues, the animal will remain in “survival mode”, which is a biological process that is designed to be rapid and intense for just a limited period of time.

However, if the stress continues, stress hormones and other pro-inflammatory molecules will remain in circulation. This will keep the animal in a chronic state of survival, oxidative stress, and chronic systemic inflammation. Furthermore, during this process, oxidative stress will damage cell and mitochondrial membranes (lipid peroxidation), which will compromise cell physiology in all tissues and organs. Chronic stress and inflammation are considered to be the “hidden killers” due to these aforementioned reasons [12,13,14].

More than a century ago, Eli Metchnikoff (1845–1916), known as the “father of innate immunity”, devised the ground-breaking concept that consuming live bacteria could improve one’s health by altering the composition of the microbiota in the intestinal tract [15,16]. For this concept, he was awarded in 1908 with the Nobel prize in Physiology or Medicine (jointly with Paul Ehrlich). The selection of antibiotic-resistant bacteria is a major concern in medical and agricultural communities across the globe. This concern has never been more pertinent, as an increasing number of antibiotic-resistant bacterial strains are posing a threat to the health of animals and humans. Resistance mechanisms have been identified and documented for all known antimicrobials that are currently available for clinical use [17].

In the interest of improving animal nutrition, also several phytogenics have been investigated for their use as feed additives. Due to the antioxidant, anti-inflammatory, antibacterial, antiviral, antifungal, immunomodulatory, and barrier-integrity-enhancing qualities of phytogenics, they play a vital part in the prevention of a number of disorders.

The administration of therapeutic and subtherapeutic levels of antimicrobials to animals is currently garnering an increased amount of interest from both the general public and the scientific community. This can be attributed to the rise and spread of a large number of antibiotic-resistant zoonotic bacterial infections. As a direct response to the pressures exerted by society, regulations that restrict the use of antibiotics in food-producing animals has been implemented. Evaluating potential antibiotic alternatives is necessary in order to achieve a food animal production system that remains highly efficient when antimicrobials are reduced. According to current research, nutritional strategies that combat the debilitating effects of stress, sickness, and chronic inflammation may in some instances prove to be good alternatives to some use of antibiotics.

It has been shown that enhancing disease resistance in animals maintained without antibiotics is beneficial to their health, welfare, and production, as well as a vital tactic in enhancing the microbiological safety of animal products. Probiotics, prebiotics, enzymes, short- and medium-chain fatty acids, and phytochemicals are just a few of the alternative feed additives that have been researched and developed as a result of recent international legislation and growing consumer demands to remove growth-promoting antibiotics and limit the therapeutic use of antimicrobials [18,19,20]. Food safety concerns and the rapid, unique use of novel research methodologies derived from systems biology (‘omics’) and biomarkers to accurately assess “gut health” will influence this field in the future. In this Special Issue, entitled “Nutraceuticals to Mitigate the Secret Killers in Animals”, we will focus on manuscripts that address the most recent advances in nutraceuticals, such as phytogenic substances, prebiotics, and probiotics, to mitigate the Secret Killers in animals. Additionally, we will highlight on the mechanisms of action, as well as the challenges and prospects of nutraceutical applications.
